# Detection of aflatoxin-producing fungi isolated from Nile tilapia and fish feed

**DOI:** 10.17179/excli2017-960

**Published:** 2017-12-13

**Authors:** Hams M.A. Mohamed, Walaa F.A. Emeish, Albert Braeuning, Seddik Hammad

**Affiliations:** 1 Department of Microbiology, Faculty of Veterinary Medicine, South Valley University, 83523-Qena, Egypt; 2 Department of Fish Diseases and Management, Faculty of Veterinary Medicine, South Valley University, 83523-Qena, Egypt; 3 Department of Food Safety, German Federal Institute for Risk Assessment, 10589-Berlin, Germany; 4 Department of Forensic Medicine and Veterinary Toxicology, Faculty of Veterinary Medicine, South Valley University, 83523-Qena, Egypt; 5 Molecular Hepatology Section, Department of Medicine II, Medical Faculty Mannheim, Heidelberg University, Mannheim, Germany

**Keywords:** Oreochromus niloticus, A. flavus, aflatoxins, clove oil

## Abstract

Contamination of fish by fungi and their mycotoxins poses major health concerns to human and animals. Therefore, our study was aimed to investigate *Aspergillus flavus (A. flavus)* infections and the levels of aflatoxins in Nile tilapia, *Oreochromis niloticus (O. niloticus)*, and fish feed. Samples from *O. niloticus* and fish feed (n=25 for each) were randomly collected from private fish farms at Qena province, Egypt, during the winter season. Different* Aspergillus* spp. were detected in 60 % and 64 % of *O. niloticus* and fish feed, respectively. HPLC-based analysis revealed aflatoxin-producing activity in 75 % and 83 % of *A. flavus* isolates from fish and fish feed, respectively. While 96 % of *O. niloticus *muscles and fish feed samples were contaminated with aflatoxins, the detected levels were below the permissible limits, i.e. 20 µg/kg. Moreover, experimental infection with toxicogenic *A. flavus* isolates was conducted to evaluate their pathogenicity in* O. niloticus*. Expectedly, experimental infections of *O. niloticus* with *A. flavus* were associated with several clinical symptoms reported in naturally infected fish, e.g. yellow coloration with skin ulceration, hemorrhagic ulcerative patches on gills and skin, corneal opacity, fin rot and abdominal distention. Furthermore, aflatoxicogenic* A. flavus* isolates from fish were sensitive to herbal clove oil. Even though the measured levels of aflatoxin were below permissible limits, effort should be placed on further reduction of exposure to genotoxic and carcinogenic mycotoxins.

## Introduction

Fish is an important source of animal protein production. With the increasing demand for fish as human food, fish farming is rapidly expanding all over the world to compensate for the deficiency in animal protein. Nile Tilapia (*Oreochromis niloticus; O. niloticus*) is considered one of the most popular fishes in Egypt, because of its cheap price and palatability. It is widely cultured because of its high growth rate, ability to withstand stress and diseases, ability to spawn easily, and the minimal requirements with regard to management and energy inputs (Nandlal and Pickering, 2004[27]). 

Fungal diseases are the second most serious cause of losses in aquaculture. Several fungi affecting fish are considered opportunistic, i.e. to attack fish when they are stressed or immune-compromised because of unfavorable environmental conditions. Aggravated susceptibility to fungal infections occurs secondarily to bacterial or viral infections, or when fish has lost mucus protection because of trauma or excessive handling (Quiniou et al., 1998[34]). *Aspergillus spp.* causes systematic diseases with high mortality rates in fish, whereby the infections mostly occur through contamination of fish feed (Urquhart et al., 2016[43], Willoughby, 1994[44]). Today, more than 400 different mycotoxins are known: the most important mycotoxins are aflatoxins, which are highly carcinogenic, cause hemorrhages, hepatotoxicity, nephrotoxicity, neurotoxicity, dermatitis, as well as immunosuppressive effects, e.g. decreased antibody and cytokine levels (Richard, 2007[37]; Shephard, 2008[42]). 

New and safe antimicrobial agents are urgently needed to prevent and overcome severe fungal aquaculture losses. Many essential oils such as clove oil have antioxidant activity and anti-fungal properties (Guynot et al., 2003[13]; Pinto et al., 2009[31]). In the present study, we investigate* Aspergillus flavus *(*A. flavus*) infections and aflatoxin contaminations in *O. niloticus* and fish feed. Further, we evaluate the pathogenicity of aflatoxicogenic *A. flavus* isolated from fish or fish feed samples along with their sensitivity to the anti-fungal agent clove oil.

## Materials and Methods

### Collection of samples

#### Fish samples 

A total of 25 Nile tilapia (*O. niloticus*), with a body weight of 70-90 g were randomly collected from 3 different private aquaculture fish farms in Qena province, Egypt, during the winter season of 2015 and identified by means of morphological analysis: tilapia belong to a group of fish called cichlids which are native to Africa. Tilapia species can be differentiated from each other by examining their body coloration and fins. Morphologically, *Oreochromus niloticus* (*O. niloticus)* is reddish to white in color with prominent bars on caudal fins and with white color strips on dorsal and anal fins (Nandlal and Pickering, 2004[27]). Collected samples were directly transferred to the laboratory on ice packs. Fish were inspected for general body condition, clinical signs and apparent lesions. Further, fish were also subjected to both mycological and mycotoxicological examination.

#### Fish feed samples

A total of 25 fish feed samples were obtained from fish farms (Al-Safwa, Assuit, Egypt). They contained the following ingredients: 23 % protein, 20 % soy bean, 20 % yellow corn, 15 % barley, 10 % wheat, 3 % carbohydrate, 2 % salt, 0.3 % vitamins and minerals. These samples were collected in sterile, clean and dry plastic bags and then transferred to the laboratory for mycological and mycotoxicological examination.

### Mycological analysis

The isolation was done according to Alniaeem et al. (2015[2]) with slight modifications as follows: briefly, Dicloran rosebengal chloramphenicol media (DRBC) were used as recommended by Samson et al. (2004[39]). DRBC contained 5 g/L peptone, 10 g/L glucose, 1 g/L KH_2_PO_4_, 0.5 g/L MgSO_4_ x 7H_2_O, 0.1 % Dicloran (0.2 % in ethanol), 0.025 g/L rosebengal, 0.1 g/L chloramphenicol, and 15 g/L agar. Subsequently, 10 g of each sample from fish (abdominal muscle) and fish feed were crushed aseptically by a mortar and mixed well with 10 mL pepton water. Of this mixture, 0.1 mL was plated on DRBC media. Plates were incubated at 25 °C for 3-7 days.

### Phenotypic identification of fungal isolates

Morphological characterization of fungi was based on the macro- and microscopic appearance of hyphae and spores of filamentous fungi according to several reports (Domsch et al., 2007[5]; Pitt, 1979[32]; Pitt and Hocking, 1997[33]). Yeast isolates were identified according to Ellis et al. (2007[6]).

### Production and extraction of aflatoxins from A. flavus isolates

Extraction of aflatoxin produced by *A. flavus* isolates was performed using synthetic medium, Yeast Extract Sucrose Broth (YES; 2 % yeast extract and 20 % sucrose). Spore suspensions of the isolates were prepared and adjusted to approximately 5x10^6 ^spores/mL by use of a hemocytometer. One mL spore suspension was inoculated into a flask with 50 mL of sterile YES and incubated at 25 °C for 7 days. After incubation, the entire contents were blended and chloroform was added to the broth (1:1) in a flask. The mixture was kept on the shaker for 24 h. Mixtures were separated in separator funnel to an upper layer containing spores and mycelia, and a lower layer containing chloroform and mycotoxins. The chloroform phase was evaporated in a water bath at 50 °C and kept in a dark dry bottle (Khaddor et al., 2007[23]).

### Extraction of aflatoxins from fish muscles 

Extraction and purification of total aflatoxins from fish muscle was carried out using the method described by Hassan et al. (2011[17]): briefly, 20 g of each sample was treated with 100 mL acetone:water (50:50 v/v). Subsequently, samples were blended at high speed for 3 minutes. The mixture was shaken for 30 minutes and filtered through a Whatman No. 1 filter paper. Purification of mixtures was done by addition of 40 mL of 10 % NaCl, 25 mL n-hexane, and finally 25 mL dichloromethane. The extracts were collected and evaporated to dry in dark bottles.

### Extraction of aflatoxins from fish feed samples 

Twenty gram of each sample was ground and homogenized for 5 minutes in a high-speed blender at 16000 rpm and mixed well with 100 mL chloroform. The chloroform extract was washed with an equal volume of distilled water, dried over anhydrous sodium sulfate, filtered, evaporated and dried in dark bottles, and the residue was used for the derivatization according to Zohri and Sabah (1992[48]).

### Quantitative estimation of aflatoxins by HPLC 

All chemicals and aflatoxins standards were purchased from Sigma-Aldrich, USA. Aflatoxins B1, B2, and G2 standards (1.0 mg of each aflatoxin in capped amber bottles) were used. The working solutions were prepared according to AOAC (2000[3]) by injecting 1mL of acetonitrile into each vial to dissolve the aflatoxins. Working solutions were stored at 8 °C.

To estimate aflatoxin levels, we applied a method described by Hassan (2003[16]), with minor modifications as follows: 100 μL of chloroform were added to the sample extract or to the aflatoxin working standards, mixed well for 30 seconds, and then filtered using a No. 4 Whatman filter paper. Then, 900 µL of water:acetonitrile (9:1 v/v) were added and mixed well for 30 seconds. Thirty microliters of this mixture were injected into a high-performance liquid chromatography (HPLC) column which had been preconditioned with 5 mL methanol and 5 mL acetonitrile:water (9:1 v/v) for 15 min. The HPLC system consisted of an Agilent Technologies Pump Model 1200 Series, G1321A FLD system. A fluorescence detector was used for the quantitation under the following conditions: FLD at 295 nm (excitation) and 330 nm (emission); Column Zorbax Eclipse Plus C18 Analytical 4.6 × 250 mm, 5-Micron; Post Column UVE LC Tech, Photochemical Post Column Derivatizer UVC 254 nm. All HPLC analyses were carried out under isocratic conditions using a mobile phase of acetonitrile:methanol:water (30:15:55 v/v/v) and the flow rate was fixed at 1.0 mL/min (see the standard curve, Supplementary Figure 1A). The mixture was filtered using a membrane filter and degassed in an ultrasonic bath for 25 min prior to use. The injected volume was 30 μL. 

### Experimental infection

Experimental infection was done with toxicogenic *A. flavus *isolate. Forty-five acclimated Nile tilapia (*O. niloticus)* with a body weight range of 100-150 g were used to conduct this experiment. The fishes were fed twice daily on a commercial floating powdered feed containing 45 % protein. The feeding rate was 3 % of their body weight. They were divided into three equal groups (n=15 fishes in each group). Each group was subdivided into three replicates of 5 fish. The first group served as a control (untreated), the second group was injected with sterile saline (sham control), and the third group was injected with *A. flavus* spores. The spore suspensions were prepared for experimental infection as follows: inocula were prepared by spreading 5 mL of sterile phosphate-buffered saline over the plates containing 7-10 day old pure cultures of *A. flavus*. The spores were harvested by gentle washing of the surface of the colonies with a sterile loop, and then transferred aseptically to sterile flasks. Spores were counted using a hemocytometer and the suspension was diluted to 9x10^4^ spores/mL of *A. flavus*. Fishes were challenged through intraperitoneal injection of 0.2 mL of this spore suspension (Refai et al., 2010[36]). Clinical signs and mortalities were recorded daily for 20 days. Moribund fishes were mycologically examined to re-isolate and identify the causative strain.

### Well diffusion assay

Crude clove oil (Al-Ahram Company, Egypt) was diluted in Dimethyl sulfoxide (DMSO) to obtain different concentrations (1 %, 2 %, 3 %, and 4 %, respectively). The oil was sterilized by filtration through 0.45 μm millipore filters (Nalgene, UK). Then, the antifungal screening test was carried out by well diffusion (Hili et al., 1997[19]), using 100 μL of suspension containing 1x10^5^ spores/mL of toxicogenic *A. flavus* isolate spread evenly on the surface of Sabouraud dextrose agar plates. Analyses were performed in inoculated agar (5 mm in diameter) in triplicates to contain 100 μl of each concentration. As a diluent control, 100 μL of DMSO were used. Fluconazole (Sigma; 1 mg/mL) was applied as a positive control for fungal growth inhibition (Pinto et al., 2009[31]). Hundred micro liters of drug poured in wells in triplicates per Petri dish, the inoculated plates were incubated at 25 °C for 3-5 days. Plates were tested for inhibition zones around the wells.

## Results

### Isolation of fungal species from fish and fish feed 

**Figure 1:**
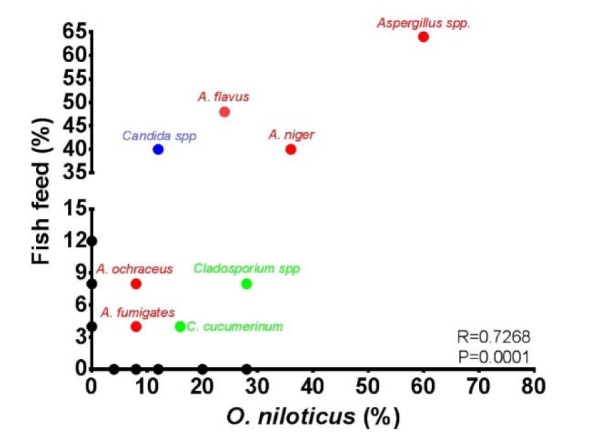
Correlation of incidences of different fungal species isolated from *O. niloticus *and feed. A significant positive correlation was calculated between the percentages of detected isolates from feed vs *O. niloticus. *All percentages were expressed in relation to the number of examined samples.

To determine the most abundant fungi in both *O. niloticus* and fish feed, phenotypic identification of fungal isolates was performed. Different fungal spp. were isolated from *O. niloticus* or fish feed. Notably, *Aspergillus spp. *were the most abundant species isolated from fish and fish feed with 60 % and 64 %, respectively (Table 1(Tab. 1), Figure 1(Fig. 1)), including* A. flavus, A. niger, A. ochraceus* and* A. fumigatus*. *Penicillium* *spp*. were the second most abundant species detected. Two genus of yeast were isolated from *O. niloticus *and fish feed, namely *Candida* and *Rhodotorula spp.*.* Candida spp*. were isolated with a varying percentage of 10 % and 40 % from of *O. niloticus* and fish feed, respectively. Several fungi were detected in feed samples only, namely *C. uredinicola, P. bervicompactum, P. corylophylum, P. camemberti, P. quercetorum,* and *P. virdicatum*. In addition, *C. herbarum, P. cyclopium, P. expansum, P. reqfortii, Botryotricum spp. *and *Rhodotorula spp. *were isolated from *O. niloticus *samples (Table 1(Tab. 1)). A strong positive correlation (R=0.7268, P=0.0001) between the frequencies of abundance of individual fungi in feed and *O. niloticus* was detected (Figure 1(Fig. 1)). This indicates that feed is an important source of fungal infections in fish farms.

### Determination of aflatoxin levels from A. flavus isolates, fish muscle and fish feed

To investigate the aflatoxicogenicity of the isolated *Aspergillus spp., *the aflatoxins B1, B2, and G2 were quantified by HPLC (Supplementary Figure 1A). We found that 9 out of 12 *A. flavus *isolates from* O. niloticus* produced different aflatoxins. Three isolates produced aflatoxin B1 at high levels, one isolate produced aflatoxin B1 at a moderate level, and three isolates produced aflatoxin B2 at a moderate level. Other isolates produced aflatoxins B1, B2, and G2 at low levels (Table 2(Tab. 2), Reference in Table 2: Zohri et al., 2014[47], Supplementary Figure 1B, Supplementary Table 1). Similarly, 10 out of 12 *A. flavus* isolates derived from fish feed produced the aflatoxins B1, B2, and G2 at low levels (Table 2(Tab. 2), Supplementary Figure 1B, Supplementary Table 2). The majority of *O. niloticus* muscles and fish feed samples (15 and 16 samples) were positive for aflatoxins B1, B2, and G2, respectively. The estimated aflatoxins were detected at low levels in approximately 96 % of the samples (Table 3(Tab. 3), References in Table 3: FAO, 1995 report[7] and FDA, 1997[9], Supplementary Figure 1C, Supplementary Table 3, Supplementary Table 4). The order of detection frequency of the three aflatoxins was aflatoxin B1>B2>G2 in both, *O. niloticus* and fish feed. However, in general, the majority of aflatoxins were detected at low levels (Table 3(Tab. 3), Supplementary Figure 1C; according to FAO 1995[7]).

### Experimental challenge using toxicogenic A. flavus isolates

**Figure 2:**
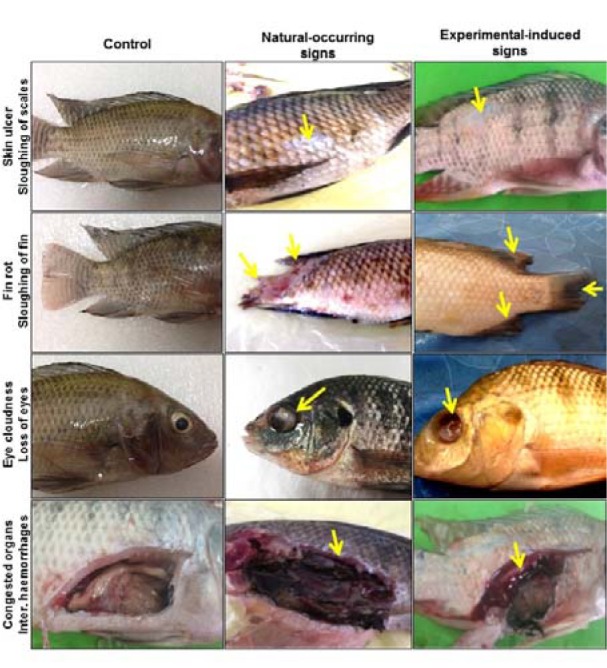
Clinical signs which were observed naturally and after experimental infection with *A. flavus*. Several symptoms were reported similarly in both naturally and experimentally *A. flavus*-infected *O. niloticus. *Among those signs were skin ulcers, sloughing of scales and caudal fin, fin rot and loss of eyes, as well as internal hemorrhages. Notably, the most frequently reported signs were more severe in case of experimentally-induced infection compared to the naturally occurring ones.

Following experimental infection of *O. niloticus* with aflatoxicogenic *A. flavus* according to the protocol detailed above, consequences of the infection were monitored in the form of observations of general clinical symptoms, e.g. loss of skin coloration with yellow body, loss of scales (Figure 2(Fig. 2), yellow arrows), sloughing of dorsal and caudal fins (Figure 2(Fig. 2), yellow arrows), moderate abdominal distention, corneal opacity, blindness, eyes covered with fungal growth (Figure 2(Fig. 2), yellow arrows), as well as internal hemorrhages (Figure 2(Fig. 2), yellow arrows). Fish of the control group appeared and acted normally with no mortality, while in the sham control group one fish died which might have been a consequence of injection stress. In the infected group, fungal growth was observed within 2 to 3 days after inoculation of *O. niloticus* with *A. flavus* and 4 fishes (26.7 %) died from the infection within 7 days. We observed the same clinical signs as for naturally infected *O. niloticus *collected from commercial fish farms (Figure 2(Fig. 2)).

### Sensitivity of toxicogenic A. flavus isolates to clove oil

**Figure 3:**
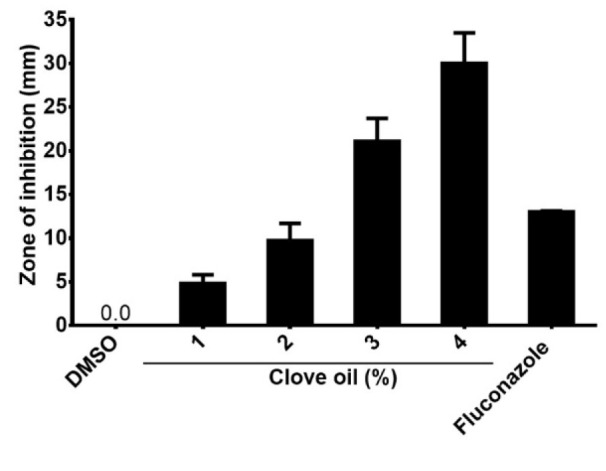
Effects of clove oil on toxicogenic *A. flavus*. The spores of *A. flavus* were isolated and incubated with different clove oil concentrations for 3-5 days and the plates were tested for inhibition zones around the wells.

Including both DMSO (negative control) and the fungicidal compound fluconazole (positive control) we measured the inhibitory zone upon exposure of *A. flavus* cultures to different concentrations of clove oil (Figure 3(Fig. 3)). Our analysis revealed a dose-dependent increase in the diameter of the inhibitory zone, indicating a remarkable anti-fungal effect of clove oil when compared with fluconazole.

## Discussion

The contamination of feed and other products by yeast and mold varies according to the geographical area, moisture, temperature, and hygienic conditions (Milićević et al., 2010[26]). Furthermore, fungal infections can be attributed to improper sanitation during catching, handling, or transportation of fish (Grace, 2015[11]; Hassan et al., 2011[17]). 

*A.* *flavus* and associated aflatoxins pose a great threat to Nile tilapia aquaculture and fish health. Here, we report that the most commonly isolated fungal species in the examined *O. niloticus* were *Aspergillus spp.* (60 %). Our findings are in agreement with previously reported data by Iqbal and Saleemi (2013[20]) in catlacatla fish. Moreover, *A. flavus* and *A. niger* represent the most common *Aspergillus spp.* isolated from *O. niloticus*. It has been stated that aspergillomycosis in African tilapia (*Oreochromis spp.*) is caused by* A. flavus, A. terreus* and* A. japonicas* (Willoughby, 1994[44]). The most common *Penicillium spp*. in our analyses was *P. expansum* (20 %), which is well in accordance with similar results by Hassan et al. (2011[18]). In addition, we detected *Cladosporium spp. *at a lower incidence as described previously by Hasballah and Abd El-Hamid (2016[14]). Isolation of yeast species was less frequent as was noticed in case of *Candida spp. *and* Rhodotorula spp., *comparable with the findings reported by Hassan et al. (2011[18]). Variations in the data are most likely due to differences in geographical area, moisture, temperature, and hygienic conditions (Milićević et al., 2010[26]).

The source of fungal infection of fish in aquaculture might be the use of contaminated feed. This hypothesis is supported by the observed correlation of fungal occurrence in fish and fish feed. Moreover, decomposition of such feed increases fungal infections (Iqbal and Saleemi, 2013[20]). These previous findings are supported by the results of the present study, as there were 3 genera of mold species and one genus of yeast identified from fish feed samples. The incidences of contamination of fish feed by *Aspergillus spp.*, especially *A. flavus* (Figure 1(Fig. 1), Table 1(Tab. 1)) which was predominantly recovered from feed samples, were similar to those reported by Almeida et al. (2011[1]) and Hassan et al. (2011[18]). Fungal load increased during the storage period of feed at high moisture levels (Kumar et al., 2017[25]). If such contaminated feed is consumed by fish, it may cause acute deleterious effects leading to massive mortality (Olufemi, 1983[30]).

Aflatoxin production in *A. flavus* is tightly regulated by growth conditions. High levels of aflatoxin synthesis require media containing easily metabolizable sugars such as sucrose, a temperature between 25 °C-30 °C, ammonia, and a nitrogen source. Therefore, fungal isolates from fish and fish feed were cultures at 25 °C on YES broth, which contains all components necessary for enhanced toxin production (Klich et al., 2009[24]; Shang et al., 2011[41]). The aflatoxicogenicity of *A. flavus* from fish, particularly aflatoxin B1 production, has been reported before (Hashem, 2011[15]; Hassan et al., 2011[18]). Toxicogenic isolates of *A. flavus* which contaminate fish feed produced aflatoxins B1, B2, and G2 at low levels. These results agree with a paper by Hassan et al. (2011[18]) who found that aflatoxins were produced at low levels by *A. flavus* isolates from fish feed samples.

It is generally accepted that not all strains of *A. flavus* are considered toxicogenic (Gerbaldo et al., 2012[10], Jaimez-Ordaz et al., 2003[22]) In agreement with that, our study reported that only some strains isolated from fish and fish feed are aflatoxin producers. The levels of aflatoxins in fish muscles and feed samples were lower than the limits reported by the FAO in 1995[7] and 2004[8] (maximum permissible limit of 20 ppb in feedstuff) (Table 3(Tab. 3), Supplementary Figure 1C). Higher values exceeding these values have been reported by Hassan et al. (2011[17][18]). We detected low levels of aflatoxins B1, B2, and G2 in fish and feed, even though we found some isolates of *A. flavus* isolated from fish which produced aflatoxins at a high level *in vitro*. The HPLC methodology used in our study is considered the most reliable and commonly used analytical technique for mycotoxin detection (FDA, 1997[9]). The discrepancy between *in vitro *and* in vivo* (fish) in aflatoxin levels can be explained by different environmental conditions e.g. favorable media. Moreover, fish might be infected with aflatoxin-producing fungi via moldy feed which is caused by high moisture content or improper storage and transport of feeds (Ramakrishna et al., 1996[35], Cagauan et al., 2004[4]; Santacroce et al., 2008[40]).

Several clinical findings have been reported after experimental exposure to toxicogenic* A. flavus*. For example, observations of sloughing of dorsal and caudal fins in our study are supported by Refai et al. (2010[36]). These clinical findings were similar in natural and experimental infection. Almost all fungi identified in this study can be categorized as normal mycoflora. However, this does not mean that they cannot cause disease. They can rather be considered opportunistic fungi, as many of them have virulence factors, which enable them to cause diseases, particularly under favorable predisposing conditions. 

Recently, it has been reported that fungal infections in fish (Yanong, 2003[46]) have increased. This might be due to 1) a lack of an effective anti-fungal therapy, 2) the development of fungicide-resistant strains (Nowakowska et al., 2009[29]), and 3) the toxicity of the most relevant anti-fungal compounds (Gupta and Tomas, 2003[12]). Therefore, there is a mandatory need to develop new strategies against aflatoxicogenic strains of *A. flavus *in fish (Gupta and Tomas, 2003[12]; Wormley and Perfect, 2005[45]). Therefore, the toxicogenic *A. flavus* isolate from fish which had been used to conduct the challenge experiment was tested for its sensitivity to clove oil. Results showed that the strain was sensitive to different concentrations of clove oil. The complete inhibition zone was formed at a concentration of 4 % (30 mm) (Figure 3(Fig. 3)). These results fit the data by NCCLS (2008[28]) and the Rosco criteria (Rosco Diagnostics, 2011[38]) which found that any inhibition zone more than 19 mm was indicative of sensitivity. Similar results have been published by Zohri et al. (2014[47]) who recorded a diameter of the inhibition zone of 36 mm. We observed that the inhibition zone was increased in its diameter from 4.8 mm to 30 mm by increasing the concentration of clove oil. This is in agreement with Jageethadevi et al. (2012[21]). The variation in the size of the inhibitory zone in well diffusion tests can be attributed to the differences in diffusion of oil in the contaminated medium with fungi under laboratory conditions and may be due to the resistance of some isolates (Wormley and Perfect, 2005[45]).

## Conclusion

Our results indicate that the detected concentrations of aflatoxin B1, B2, and G2 in *O. niloticus* and fish feed samples from the Egyptian market do not exceed international permissible limits as reported by FAO (1995[7]). As aflatoxins are genotoxic and carcinogenic food and feed contaminants, further effort should be placed on continued reduction of aflatoxin in order to minimize health risks for consumers and farmed animals.

## Acknowledgement

The authors thank the members of the analytical chemistry unit at Assiut University for their efforts in the estimation of aflatoxin levels in fish and fish feed by HPLC.

## Supplementary Material

Supplementary data

## Figures and Tables

**Table 1:**
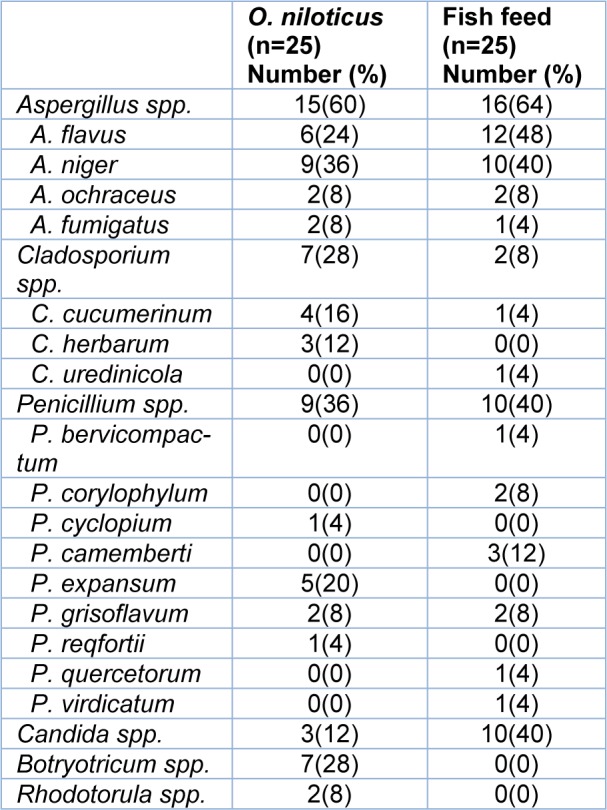
Different fungal species isolated from *O. niloticus *and feed. Out of 25 samples from *O. niloticus* and fish feed, several isolates were detected by phenotypic characterizations. *Aspergillus spp.* were the most abundant fungi in both, *O. niloticus* and feed.

**Table 2:**
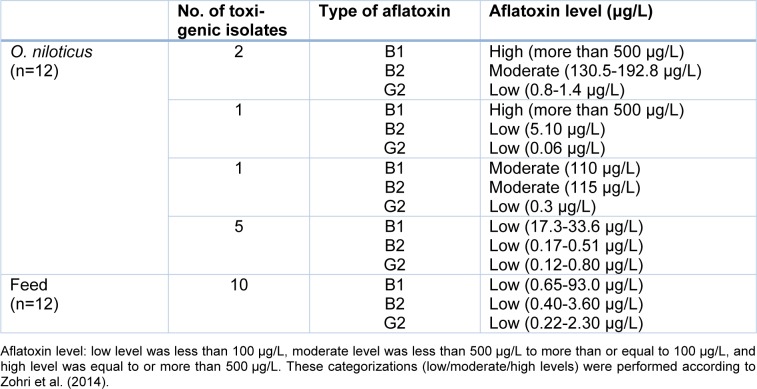
Aflatoxicogenicity of *A. flavus* strains isolated from *O. niloticus* and fish feed samples

**Table 3:**
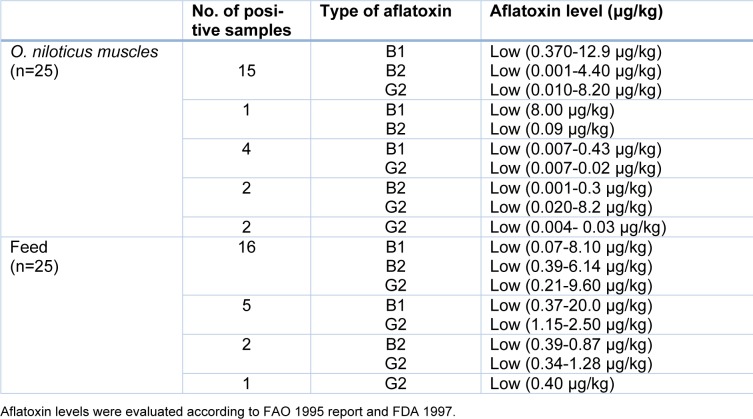
Natural occurrence of aflatoxins in muscles of *O. niloticus* and fish feed samples.
