# Supplementary material for: Detection of aflatoxin-producing fungi isolated from Nile tilapia and fish feed
Source: EXCLI J. 2017 Dec 13;16:1308–18. doi: 10.17179/excli2017-960 (PMC5763081; doi:10.17179/excli2017-960)
Supplement: Supplementary data [file EXCLI-16-1308-s-001.pdf]

EXCLI Journal 2017;16:1308-1318 – ISSN 1611-2156
Received: November 07, 2017, accepted: December 11, 2017, published: December 13, 2017

Supplementary data to:

DETECTION OF AFLATOXIN-PRODUCING FUNGI ISOLATED FROM NILE TILAPIA AND FISH FEED

Hams M.A. Mohamed[1], Walaa F.A. Emeish[2], Albert Braeuning[3], Seddik Hammad[4,5*]

[1] Department of Microbiology, Faculty of Veterinary Medicine, South Valley University, 83523-Qena, Egypt
[2] Department of Fish Diseases and Management, Faculty of Veterinary Medicine, South Valley University, 83523-Qena, Egypt
[3] Department of Food Safety, German Federal Institute for Risk Assessment, 10589-Berlin, Germany
[4] Department of Forensic Medicine and Veterinary Toxicology, Faculty of Veterinary Medicine, South Valley University, 83523-Qena, Egypt
[5] Molecular Hepatology Section, Department of Medicine II, Medical Faculty Mannheim, Heidelberg University, Mannheim, Germany

* Corresponding author: Dr. Seddik Hammad, Tel.: 00496213835603,
E-mail: seddik.hammad@vet.svu.edu.eg

http://dx.doi.org/10.17179/excli2017-960

This is an Open Access article distributed under the terms of the Creative Commons Attribution License (http://creativecommons.org/licenses/by/4.0/).

EXCLI Journal 2017;16:1308-1318 – ISSN 1611-2156
Received: November 07, 2017, accepted: December 11, 2017, published: December 13, 2017

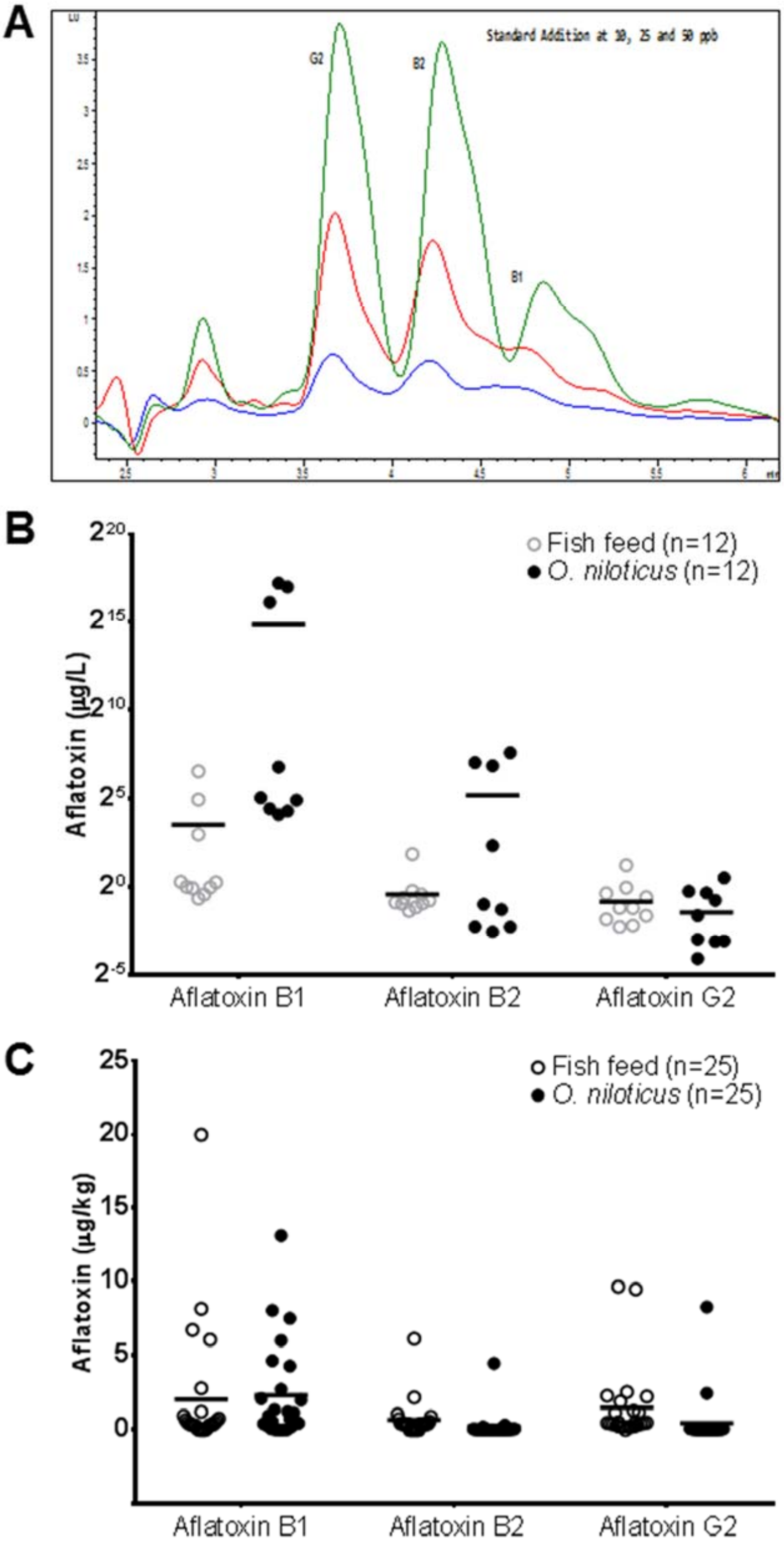

Supplementary Figure 1: A) The levels of aflatoxins in *O. niloticus* samples and feed were detected by HPLC according to the standard curve. **B)** and **C)** levels of aflatoxin B1, B2, and G2 were estimated in *A. flavus* isolates, farm-collected *O. nilotcus* and feed samples. Black lines refer to means.

EXCLI Journal 2017;16:1308-1318 – ISSN 1611-2156
Received: November 07, 2017, accepted: December 11, 2017, published: December 13, 2017

Supplementary Table 1: Aflatoxins secreted by *A. flavus* isolates of fish samples

***A. flavus* isolates of fish**	**Concentration of Aflatoxins (µg/L)**		
	**Aflatoxin G2**	**Aflatoxin G2**	**Aflatoxin G2**
**1**	1.454	192.818	153070.684
**2**	0.000	0.000	0.000
**3**	0.128	0.173	17.381
**4**	0.804	0.421	22.016
**5**	0.000	0.000	0.000
**6**	0.117	0.212	30.604
**7**	0.000	0.000	0.000
**8**	0.060	5.148	72190.035
**9**	0.856	130.573	131981.460
**10**	0.604	0.211	20.014
**11**	0.120	0.515	33.604
**12**	0.331	115.455	110.17

Supplementary Table 2: Different aflatoxins secreted by *A. flavus* isolates in fish feed

***A. flavus* iso-lates in fish feed**	**Concentration of Aflatoxins (µg/L)**		
	**Aflatoxin G2**	**Aflatoxin B2**	**Aflatoxin B1**
**1**	0.450	0.530	0.650
**2**	0.682	0.661	0.760
**3**	0.992	0.871	1.001
**4**	0.000	0.000	0.000
**5**	0.792	0.761	0.970
**6**	0.331	3.695	93.170
**7**	0.446	0.601	1.246
**8**	0.212	0.538	31.434
**9**	0.000	0.000	0.000
**10**	2.390	0.459	8.051
**11**	0.224	0.401	1.025
**12**	0.287	0.556	1.222

EXCLI Journal 2017;16:1308-1318 – ISSN 1611-2156
Received: November 07, 2017, accepted: December 11, 2017, published: December 13, 2017

Supplementary Table 3: Detected aflatoxins in fish samples

Sample	Concentration of Aflatoxins (µg/Kg)		
	Aflatoxin G2	Aflatoxin B2	Aflatoxin B1
1	0.019	0.013	0.549
2	0.017	0.008	1.133
3	0.008	0.003	0.179
4	0.055	0.002	0.907
5	0.008	0.019	1.344
6	0.068	0.006	1.192
7	0.009	0.000	0.271
8	0.075	0.024	4.608
9	0.038	0.054	7.468
10	0.004	0.000	0.000
11	0.010	0.000	0.070
12	0.031	0.022	1.986
13	0.007	0.000	0.439
14	0.047	0.150	2.636
15	0.030	0.000	0.000
16	0.001	0.058	0.444
17	0.091	0.193	12.988
18	0.054	0.001	2.083
19	0.021	0.000	0.382
20	0.024	0.001	0.000
21	0.006	0.049	0.379
22	0.000	0.000	0.000
23	2.470	4.480	2.550
24	8.280	0.320	0.000
25	0.000	0.090	8.000

EXCLI Journal 2017;16:1308-1318 – ISSN 1611-2156
Received: November 07, 2017, accepted: December 11, 2017, published: December 13, 2017

Supplementary Table 4: Aflatoxins in fish feed samples

Fish food samples	Concentration of Aflatoxins (µg/Kg)		
	Aflatoxin G2	Aflatoxin B2	Aflatoxin B1
**1**	1.182	0.400	0.621
**2**	1.287	0.877	0.000
**3**	1.156	0.000	0.675
**4**	0.444	1.046	1.222
**5**	0.215	0.396	0.299
**6**	0.488	0.398	8.160
**7**	0.548	0.396	0.366
**8**	2.303	0.000	0.393
**9**	2.555	0.000	20.00
**10**	0.418	0.418	6.084
**11**	2.251	0.395	0.500
**12**	0.249	0.716	2.806
**13**	0.212	0.396	0.479
**14**	0.322	0.395	0.322
**15**	0.427	0.000	0.000
**16**	0.210	0.396	0.298
**17**	0.349	0.395	0.000
**18**	0.470	0.447	0.948
**19**	0.514	0.000	0.364
**20**	0.240	0.404	6.740
**21**	1.921	0.573	0.355
**22**	0.409	0.000	0.763
**23**	9.470	6.14	0.070
**24**	9.650	2.19	0.125
**25**	0.000	0.000	0.000
